# A scoping review of digital fabrication techniques applied to prosthetics & orthotics: Part 2 of 2—orthotics

**DOI:** 10.1097/PXR.0000000000000399

**Published:** 2024-11-13

**Authors:** Ben M. Oldfrey, Dafne Z. Morgado Ramirez, Catherine Holloway, Matthew Wassall, Christopher Nester, Alex Dickinson, Man S. Wong, Jamie Danemayer, Laurence Kenney, Edward Lemaire, Nerrolyn Ramstrand, Hossein Gholizadeh, Laura E. Diment, Margaret K. Donovan-Hall, Mark Miodownik

**Affiliations:** 1 Global Disability Innovation Hub (GDI Hub), London, United Kingdom; 2 Institute of Making, University College London, London, United Kingdom; 3 University College London Interaction Centre (UCLIC), London, United Kingdom; 4 Centre for Human Movement and Rehabilitation Research, University of Salford, Salford, United Kingdom; 5 School of Allied Health Professions, Keele University, Keele, Staffordshire, United Kingdom; 6 Faculty of Engineering & Physical Science, University of Southampton, Southampton, United Kingdom; 7 Department of Biomedical Engineering, The Hong Kong Polytechnic University, Kowloon, Hong Kong; 8 Faculty of Medicine, University of Ottawa, Ottawa Hospital Research Institute, Ottawa, ON, Canada; 9 CHILD Research Group, Department of Rehabilitation, School of Health and Welfare, Jönköping University, Jönköping, Sweden; 10 College of Science and Engineering, Flinders University, Tonsley, Australia; 11 Faculty of Health Sciences, University of Southampton, Southampton, United Kingdom

**Keywords:** orthotics, digital fabrication, CAD/CAM, additive manufacture

## Abstract

**Introduction:**

Traditionally, orthosis manufacturing is time and labor-intensive. Digitalization of some of the fabrication process is already ubiquitous, yet extension across device types could reduce the burden of manual labor and advance automation to help unblock access to assistive technologies globally. It seems, however, that appropriately strong evidence is holding this back. This review looks to thoroughly examine the current state of evidence to make this clear.

**Objectives:**

To identify gaps in the literature that create barriers to decision-making on either appropriate uptake by clinical teams or setting research directions, by identifying what forms of evidence the current research literature provides to the orthotics community.

**Study design:**

Scoping literature review.

**Methods:**

A comprehensive search was completed in the following databases: AMED, MEDLINE, EMBASE, Global Health Archive, CINAHL Plus, Cochrane Library, Web of Science, ACM, IEEE, and Engineering Village, resulting in 3487 articles to be screened.

**Results:**

After screening, 121 lower limb orthosis, 104 upper limb orthosis, and 30 spinal orthosis articles were included in this review. For some areas such as CAD/CAM-produced insoles and spinal orthoses, the evidence base is strong. For most additive manufacture articles, long-term, larger-scale studies as well as research into training requirements are lacking.

**Conclusion:**

The advantages of digital fabrication technology that could streamline orthotic device production in many cases are still blocked by a lack of strong formal evidence, ie large longitudinal studies with a range of evaluation measures. Increased collaboration between clinicians, patient/service users, academia, and industry could be a route to addressing these gaps and creating a better pathway to market for new technologies.

## Abstract 2

Supplemental Digital Content is Available in the Text.

## Introduction

Approximately 0.5% of the global population requires prostheses, orthoses, and rehabilitation treatment. This estimate corresponds to 35-40 million people, and the need is expected to double by 2050.^1^ The World Health Organization (WHO) estimates that only 5-15% of people who could benefit from assistive products have access to appropriate devices,^2^ including prostheses and orthoses. One possible route to improving device access and quality is digital technologies, with the number of commercial digital fabrication offerings multiplying every year. As the second of 2 papers, with the first focusing on prosthetics, a scoping review of digital technologies is presented as applied to orthosis fabrication. This review aims to understand whether the research literature has the necessary forms of evidence to enable evidence-based decisions on either appropriate clinical uptake or further development. Where this is not the case, the aim is to understand study design limitations and make recommendations to guide future research planning.

Orthoses are assistive devices that exert external forces on parts of the body to support joints, correct deformity, or protect injuries while they heal.^3^ This ranges from minor finger positioning, joint support, and spinal bracing to full body actuating exoskeleton devices^4,5^ (Section 3.1).

Traditionally, orthosis manufacturing can involve varying pathways, depending on the application. In this review, we were particularly concerned with orthoses that are created with a high degree of individual customization, as opposed to mass manufactured off-the-shelf orthoses, such as basic insoles, ankle, knee, or other joint supports or bracing. Digital fabrication could provide efficiency and quality improvements for these customization processes.^6^ The available evidence for digital fabrication in orthotics is highly variable across device types, as will become clear in this review.

**Figure 1.**
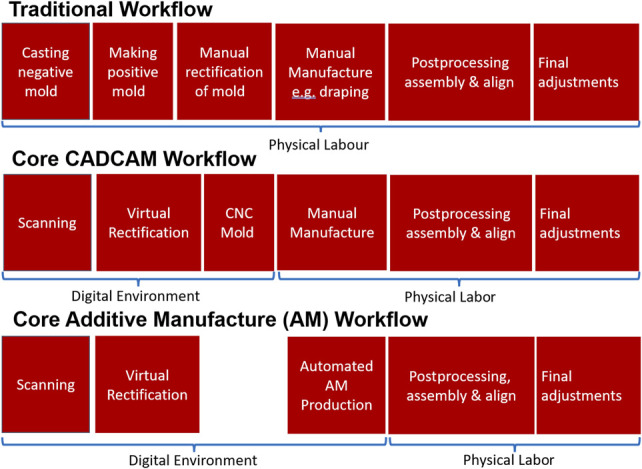
Traditional and core CAD/CAM and AM workflows for device fabrication, with all processes assumed to be clinician-led, unless CAM or AM production is outsourced.

Customized orthoses are required for a number of conditions; for example, ankle foot orthoses (AFOs) to manage walking difficulties in people with neuromuscular, musculoskeletal, and cerebrovascular conditions^7,8^ or Thoracic-Lumbar-Sacral Orthoses (TLSOs) for people with various forms of scoliosis.^9^ Traditional manufacturing involves plaster casting the body part to obtain the limb shape for device design, requiring highly skilled orthotists and technicians, dedicated plaster facilities, and many consumables.^7^ A negative plaster cast of the patient is created manually, and then a positive cast is prepared by filling the negative cast with plaster, followed by removal or addition of further material to rectify the mold based on biomechanical and clinical principles.^6^ Excessive waiting periods for children could lead to them outgrowing their devices more quickly, and limited design choices have been found to lead to user dissatisfaction and negative feelings related to use and appearance.^7,10-12^ This traditional workflow is captured in Figure 1, which also captures digitally enhanced workflows. A key benefit to digitalizing and automating the fabrication process may be a reduction in workflow time, helping to unblock access to assistive technologies globally, since improved efficiency could enable more people to be seen by the limited number of orthotists globally.^6^ As opposed to other industries, automation looks to unburden the existing orthotist community, rather than replace them, along with digital data bringing increased opportunity to learn from prior clinical datasets, although there is no clear research on this learning opportunity currently.

Digital technologies were introduced for manufacturing prostheses and orthoses as far back as the 1970s,^13^ and CAD/CAM tools enable professionals to minimize hand casting of devices. Use statistics were already high back in the 1990s for spinal orthoses. Wong et al (2006) discuss this, stating that the average number of spinal orthoses fabricated each year using the conventional method (1995 to 1998) was 113 and the CAD/CAM method (1999 to 2002) was 424 at the Prince of Wales Hospital.^6^ With this digital method, the relevant body part was scanned and creation and rectification of the positive model was replaced by digital rectification software and Computer Numerical Controlled (CNC) carving to manufacture the positive model in structural foam, upon which the orthosis is manufactured.

In recent years, there has been an increase in research that could extend the role played by digital technologies in orthosis design and fabrication. Additive manufacturing (AM)-based fabrication comprises several methods, generally based around digital scanning the relevant body part, CAD-based modeling and rectification, additive manufacturing the orthosis directly, and device-specific postprocessing. A plethora of different technologies are available for each of these stages, enabling service delivery models applicable to different contexts. This literature review covers all combinations of the digital fabrication processes of CAD/CAM and AM, along with traditional processes when these are combined with digital elements. For a full understanding of the types of AM being utilized across the articles screened in this review, and their different pros and cons, in particular—Fused Deposition Modeling (FDM) Stereolithography (SLA) and Selective Laser Sintering (SLS)—we suggest the following recent articles—Praveena et al (2022), Jadhav et al (2022), and Bhatia et al (2023).^14-16^

A consortium was assembled incorporating International Society of Prosthetics and Orthotics (ISPO) representatives, industry, and academic partners to gather evidence on digital fabrication approaches for prostheses and orthoses as part of a process of consensus building, and identify whether the necessary forms of evidence for these technologies are being achieved in the literature. In particular, the aim was to identify literature gaps that could create barriers to decision-making on either appropriate uptake by clinical teams or defining the next steps in research. The research questions for this review are as follows:

- In terms of study formulation, what are the forms of evidence that the current research literature provides to the orthotics community?
- What are the gaps in the available research that are creating a barrier to the progression of digital fabrication methods of orthotic devices?

To aid the reader, a list of acronyms used in this article are given in Appendix 1, http://links.lww.com/POI/A273.

## Methods

**Figure 2.**
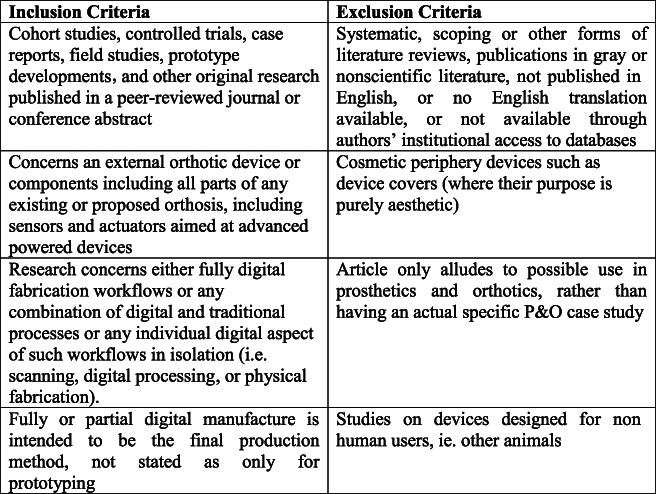
Eligibility criteria.

Studies were included based on the inclusion and exclusion criteria detailed in Figure 2, which were devised to identify appropriate original research on digital fabrication of orthotic devices. Searches were completed in July 2021, with some additional articles added in November 2021.

### Eligibility criteria

The eligibility was decided to be all types of original research across study types excluding other reviews, which concerns an external orthotic device or component. The research must concern either fully digital fabrication workflows, a combination of digital and traditional processes, or specific individual digital aspects of such workflows. For a full breakdown of the eligibility criteria, please see Figure 2.

### Information sources*

Articles were searched in 10 databases: Allied and Complementary Medicine Database (AMED), MEDLINE, Embase, Global Health Archive, CINAHL Plus, Cochrane Library, Web of Science, Association for Computing Machinery (ACM), Institute of Electrical and Electronics Engineers (IEEE) Explore, and Engineering Village.

### Search strategy

The search strings were designed to identify all articles concerning digital fabrication of external prosthetic and orthotic devices. The strings and protocols were developed iteratively with reference to a known set of expected articles and refined with Boolean operators and wild cards to limit the number of excluded articles, particularly those concerning internal and dental prostheses. The search terms were, therefore, of the format [keywords related to prostheses] AND [keywords related to digital fabrication] NOT [excluded prosthesis types, in particular dental]—an example string is given in Appendix 2, http://links.lww.com/POI/A273. Searches were conducted on all timestamps up to and including July 28, 2021. Manual reference lists and Google Scholar citation searches were completed to identify additional articles.

### Selection process

Articles were imported into the Endnote citation software.^17^ After deduplication using the Bramer method,^18^ the titles and abstracts of the remaining articles were imported into Rayyan.^19^ A broad screening review was conducted to include or exclude each article based on the title and abstract using the aforementioned criteria, with at least 2 investigators screening each article. All investigators were blind to other’s decisions until after all decisions were completed.

At least 2 investigators then reviewed each included full text and classified the article within a device category to facilitate analysis. During review and appraisal, conflicting decisions were discussed by the 2 deciding investigators, with a third investigator breaking ties if a decision could not be reached. Finally, an additional request for missing articles to be identified was made on November 1, 2021 at the ISPO World Congress, where the database list was made public, and people were invited to point out articles that should have been included. These articles were then screened after the original procedure outlined above and added to the set.

### Data collection process

A subset of articles was used to develop the method and guidelines on data field extraction, before full dataset extraction and tabulation in Excel.

### Data items

This review examines various study features in the Results section. Below is a brief description of these features and the data items extracted:

#### Distribution of papers by device type

- Device-body position or device type the study investigates.

#### Digital manufacturing process

- Specific digital manufacturing process employed in the article. Where no manufacturing took place, the premanufacturing digital process such as “Scanning Only” or “Modeling Only” was indicated.

#### Primary process focus + digital workflow

- Primary Process Focus refers to whether there is a clear primary focus of the study; for example, modeling or specifically 3D scanning.
- Digital Workflow describes the full set of stages that took place to fabricate a device.

#### Technical readiness level

**Figure 3.**
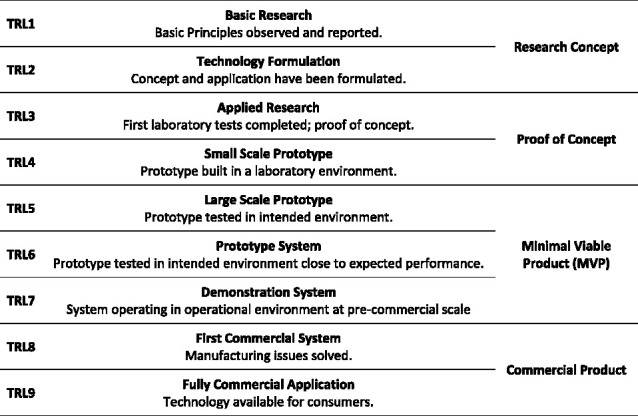
NASA TRL Scale Adapted to Assistive Technology, data taken from WIPO (2021).^20^

- NASA Technology Readiness Level (TRL) Scale was adapted to assistive technology (Figure 3)^20^ to rate the maturity of technologies being presented.

#### Sample size

- The number of participants involved in the study.

#### Qualitative methods

- Whether qualitative evaluation was undertaken, whether data were collected for more or less than a week, and the nature of the evaluation in the following categories: “Off Patient” (discussion of the produced device/prototype without patient involvement) or “On Patient” (evaluation/feedback or observational analysis of a fitted patient).

#### Quantitative methods

- Whether quantitative evaluation was undertaken, whether data were collected for more or less than a week postfitting, and the nature of the evaluation in the following categories: “Off Patient Mechanical” (mechanical testing the device without patient involvement, such as ISO equivalent,^21^ structural/material testing), “Off Patient Computational Modeling” (eg, finite element analysis), and “On Patient” (eg, quantitative gait analysis and instrumented data collection of fitted patient).

#### Materials used

- The materials used for the digitally fabricated components in the study and if the component is made indirectly (eg, a digitally created mold to cast a component in another material). This only refers to the final component’s material.

#### Chronology of submissions

- The date the article was published.

## Results

### Study selection

**Figure 4.**
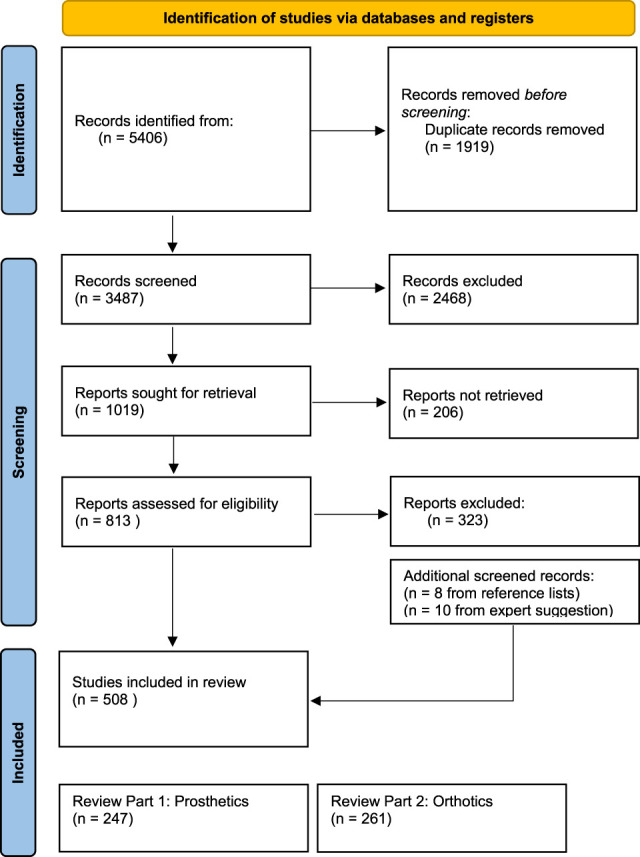
PRISMA diagram of search protocol.

The articles were split into device categories with 121 lower limb orthosis (LLO), 104 upper limb orthosis (ULO) and 30 spinal orthosis (SO) screened articles included in the review. To note, 6 cranial orthosis articles were also identified; however, with N = 0 or 1 for these studies, the category was not carried forward for analysis. Figure 4 overviews the articles across device types. In Appendix 3, http://links.lww.com/POI/A273 can be seen an overview of the specific articles discussed in the following review across device types.

#### Distribution of papers by device type

**Figure 5.**
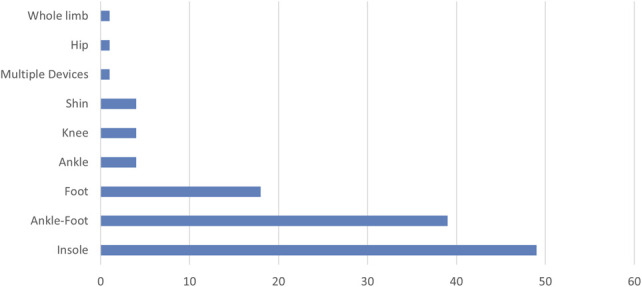
LLO article distribution by device/component types.

**Figure 6.**
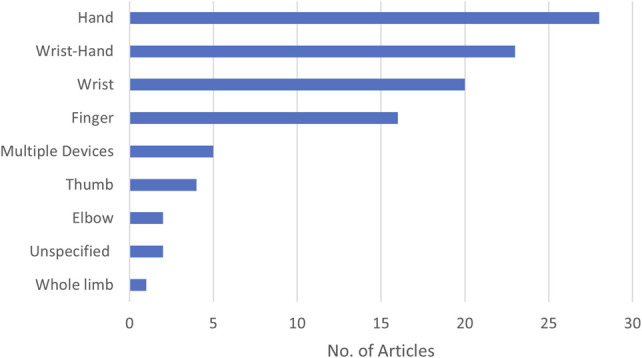
ULO article distribution by device/component types.

**Figure 7.**
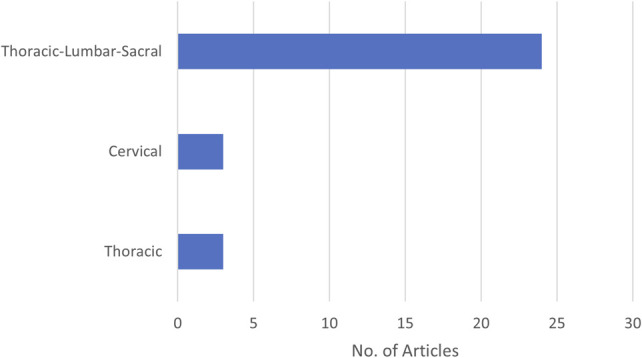
SO article distribution by device/component types.

In Figures 5–7 the distribution of articles by device type can be seen. The LLO literature was dominated by insoles and AFOs, with insoles in particular contributing some of the most mature commercialized digital technologies to be found across orthoses as a whole, with a range of larger scale trials (10 insole articles with more than 25 participants). This is expected because insoles can be produced with simpler CNC machines and the insole market is largest of all orthosis categories (ie, easier patient recruitment and more providers since insoles provision is not restricted to certified orthotists).

The application of digital fabrication for ULOs primarily concerns the hands, wrist, and fingers. Hand orthoses, particularly those with complex constructions, can be efficiently prototyped using a single AM machine, whether or not customization is needed. Wrist orthoses are a well-researched area, with a broad range of articles deploying limb scanning to produce individualized braces and shorter-term casts, with results indicating positive outcomes in comparison to traditionally fabricated or mass-produced alternatives. The scale of finger and thumb devices is also well suited to low-mid priced AM machines with a range of individualization achieved.

For SOs, TLSOs cover nearly the whole body of articles, with a few cervical collar studies being identified.

#### Major digital manufacturing process

**Figure 8.**
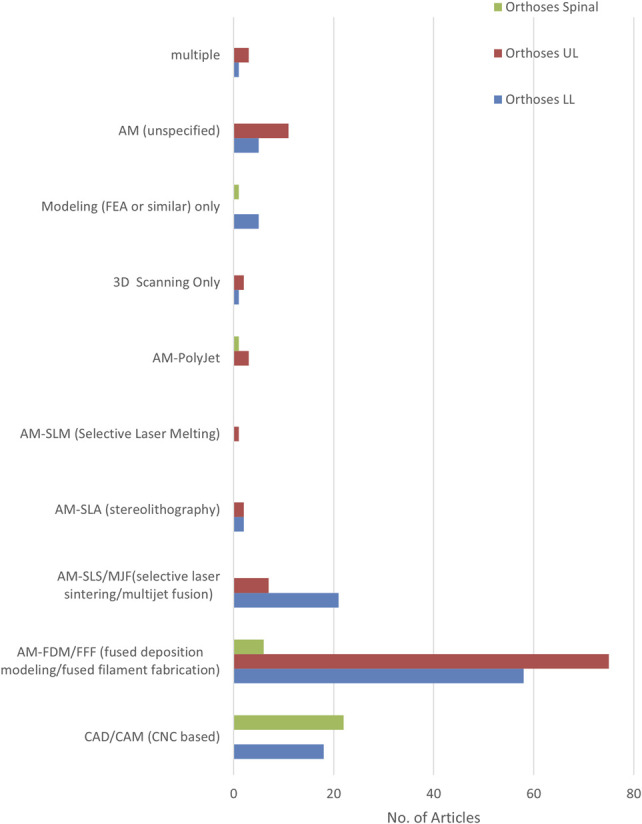
Digital manufacturing processes employed by LLO, ULO, and SO articles.

Overall, FDM dominates the literature (Figure 8), particularly for ULO where there were no CAD/CAM use found and only 11 articles using powder- or resin-based AM. For LLO, a substantial number of articles employed powder-based AM techniques for both AFOs and insoles. There were a substantial number of CAD/CAM articles, 18 on LLO, of which 17 described insoles. For SO, which had proportionally far fewer articles compared to ULO and LLO, CAD/CAM dominated the literature. CAD/CAM applied to insoles or TLSOs had multiple large scale longitudinal trials, contributing potentially strong evidence for decision-making.

#### Primary process focus + digital workflows

**Figure 9.**
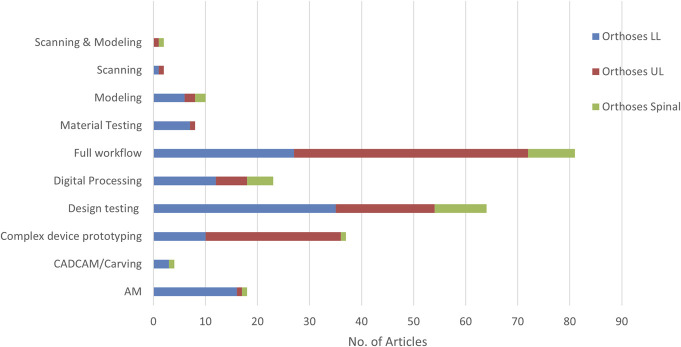
Primary focus of articles for LLO, ULO, and SO. Man = Manual anatomical data collection; Anat = Complex anatomical data collection; ie, scanning Comp = Scan of a component; CAD = Digital design altered with manual CAD work; CADClin = Digital design altered with clinically informed manual CAD; U = Unspecified digital design alteration; FEA = Finite Element Analysis used to optimize final design; CL = Machine learning or some data science method used to optimize design; CNC = Computer Numerical Controlled Carving; AM = Automated Additive Manufacture; M = Manual Fabrication; P = Postprocessing, assembly and addition (with alignment) of other components; A = Final adjustments/corrections before and during gait or equivalent training until comfortable.

The intended primary process focus of the articles was categorized, while cognizant that generally all elements of a fabrication workflow are at least summarized in most articles since they are necessary to produce a testable device. A large portion of articles were designated as design testing (ie, research focused on the physical design of a product, including a subcomponent of a device) or complex device prototyping (ie, prototypes comprising construction of many multiple parts; for example, exoskeleton devices) (seen in Figure 9). Articles that describe a full workflow were the largest portion of the literature (ie, no single process dominated the narrative). The range of these workflows is large, with varying incorporation of manual and traditional methods alongside digital processes.

**Figure 10.**
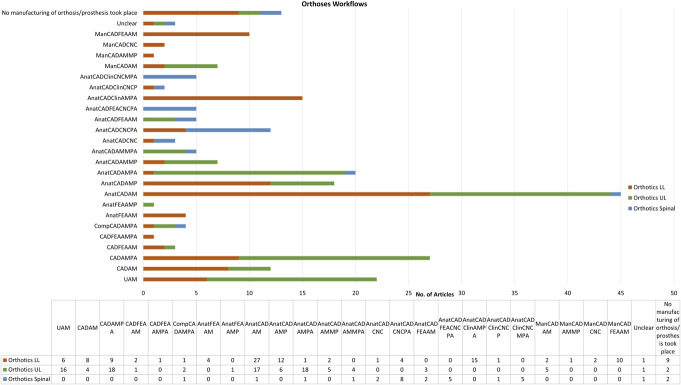
Prevalence of categorized digital workflows used in articles for LLO, ULO, and SO.

Both within core workflows of AM and CAD/CAM (Figure 1) and more broadly across the literature, a wide range of combinations of different processes are used at each distinct production phase. To understand the prevalence of different approaches, workflows within the literature were mapped, with 24 different process groupings defined for orthotics (seen in Figure 10, with a key for the process groupings given in Appendix 4, http://links.lww.com/POI/A273).

The largest portion of articles for LLO and ULO were designated **A**nat**C**AD**A**M (anatomical measurement usually 3D scanning, rectified in CAD, and AM produced), since there was little to no documentation of critical postprocessing or device adjustments. However, the second most prevalent LLO designation was **A**nat**C**AD**C**lin**A**M**PA** (anatomical measurement usually 3D scanning, clinically informed rectification in CAD, fabricated using AM, followed by some manual fabrication processes, postprocessing, and adjustments).

To note, for LLO and ULO, but not for SO, a substantial number of articles were designated **C**AD**A**M**PA**, **C**AD**A**M, and **UA**M that do not have any anatomical measurement before design or no description of the design process. Although this could be a limitation in terms of approach and evidence, these are nearly all articles at a very early stage of development and does not present a barrier to being very useful research on proof-of-concept designs.

Most SO articles had detailed descriptions of the full workflow, including postprocessing and adjustments, with designations spread mainly across **A**nat**C**AD**C**lin**C**NC**MPA** (anatomical measurement usually 3D scanning, clinically informed rectification in CAD, fabricated using CNC followed by some manual fabrication processes, postprocessing, and adjustments); **A**nat**C**AD**F**EA**C**NC**PA**, which uses finite element analysis to inform the rectification of the design; and **A**nat**C**AD**C**NC**P**A, which has no described clinical input into rectification and no description of additional manual fabrication elements, which may or may not have been present.

Across all of these workflows, the reader could assume that all the steps are undertaken, but these are often not described fully in the literature, in particular for example, descriptions of various rectifications made to designs and necessary finishing processes. Since every step in the process could affect the outcome, the lack of these full descriptions in the literature is problematic. With often limited journal word counts, authors may have no choice but to exclude information on these steps.

#### TRL level

**Figure 11.**
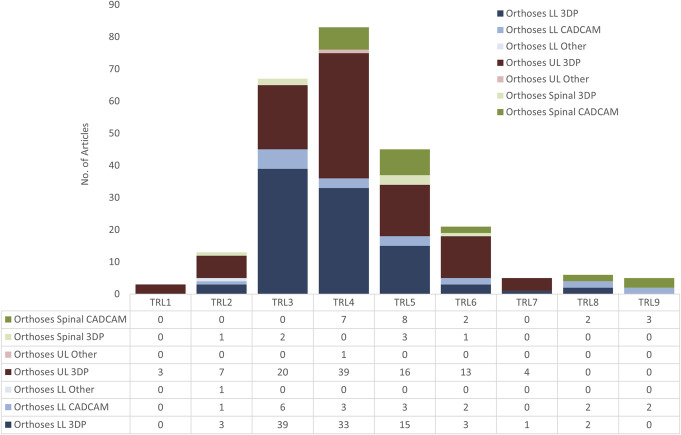
Number of LLO, ULO, and SO articles per Technology Readiness Level (TRL).

Each technology project was evaluated against the parameters for each technology level and then assigned a TRL rating (shown in Figure 3) based on the project’s progress. The results can be seen in Figure 11.

For LLO 3DP, 6 studies were designated as TRL6 and above and 4 of these had cohorts greater than 30, all with long-term quantitative and qualitative data collected. Xu et al conducted insole trials on 80 people with symptomatic flatfoot^22^ and 60 people with plantar fasciitis.^23^ These studies used similar insole technology, commercially available pressure scanning, and Ethylene-Vinyl Acetate-based AM techniques, with the former being considered a single-blind, randomized controlled study. Two studies were TRL6. Thirty-four workers used insoles for decreasing foot pain for prolonged standing, over 3 weeks of insole use, with significant positive results in favor of the new technology.^24^ Salles et al^25^ assessed AM-personalized insoles designed for running, with 38 and people using the device for over 3 months.

For LL CAD/CAM, 4 studies out of 19 were designated TRL8/9, mainly showing the maturity of insole technology; however, only one study was longitudinal (evaluation up to 12 months) and was the largest trial with N = 178,^26^ finding that CAD/CAM technology produced insoles that were more effective in preventing recurrent ulcers because of leprosy, and patients scored the insoles higher on a range of metrics. Two lab-based investigations into plantar pressure distribution of CAD/CAM vs. conventionally fabricated insoles did not produce significant differences between groups.^27,28^ Roberts et al^29^ conducted a N = 136 randomized control trial (RCT) comparing traditional casting with scan-based CAD/CAM. They found no significant improvement in quality, cost, or time to delivery, and a higher incidence of fit problems with the CAD/CAM insoles.^29^

For SO 3DP, the study deemed most mature was at TRL 6, looking at cervical collars for improving neck posture in 41 healthy participants during smartphone usage.^30^

For UL 3DP, the most matured technology studies were at TRL7 with 2 studies deemed to be in this bracket. A large number of studies were at TRL5 (16) or TRL6 (13), which were also tested outside the laboratory.

For TRL7, Chen et al^31^ conducted an N = 60 study comparing SLS-printed Nylon casts for forearm fractures using finite element analysis (FEA) modeling and computed tomography data vs. plaster cast and splint fixation as control groups, with 20 patients in each group. Outcomes on clinical efficacy, wrist function, and patient satisfaction were scored and compared up to 3 months, with the printed group scores totaling higher than the other 2 groups. This trial was predated by the same group in 2017,^32^ with a smaller, shorter TRL6 N = 10 study with 6-week follow-up and patient satisfaction questionnaires; however, this initial study did not involve FEA modeling. This is a good example of considered expansion of research protocol.

Zheng et al^33^ conducted an N = 40 RCT on the effects of an SLA-printed orthosis compared to a low-temperature thermoplastic plate orthosis on wrist flexor spasticity in patients who have had a chronic hemiparetic stroke. This 6-week comparative study using a variety of quantitative scoring approaches, showing greater change using the printed orthoses in reducing spasticity and swelling, and improving motor function of the wrist and passive range of wrist extension for patients who have had a stroke.

For SO CAD/CAM, 6 studies were deemed TRL 8/9. For the 3 TRL 9 studies, one study compared CAD/CAM TLSO with standard cast orthosis for 10 patients, with improved curve corrections over 3 months and with 78% of patients preferring the CAD/CAM orthosis.^34^ Another prospective controlled cohort observational study of 225 patients^35^ discussed the new Lyon brace or ARTbrace, an immediate corrective brace based on the same principles of previous plaster cast braces but using CAD/CAM and OrtenShape proprietary software. The article includes an in-detail description of the design process and theory of action. The ARTbrace had better reduction rates and improved aesthetic appearance. D'Amato et al^36^ (2001) conducted an N = 102 trial on specifically female adolescent idiopathic scoliosis (AIS) patients, with excellent in-brace correction observed with the CAD/CAM made Providence braces.

At TRL 8, Wong et al^37^ conducted a comparative trial on 147 people with AIS. Forty-three people had the conventional method and 104 had the CAD/CAM method. Cobb angle, apical vertebral rotation, and trunk listing were measured at prebrace, 4, 8, and 12 months postbrace provision, along with an investigation of the reducing worktime of orthotists for different processes involved in the CAD/CAM method.

Guy et al (2021)^38^ conducted a TRL 8 single-center prospective randomized controlled trial also looking at AIS with N = 120, with 94 completing the whole study of 2 years.^38^ The study compared orthoses designed using CAD/CAM with and without patient-specific FEM simulations of the spine, rib cage, and pelvis. Results were not significantly different, with satisfying outcomes in both groups.^38^

### Sample size

**Figure 12.**
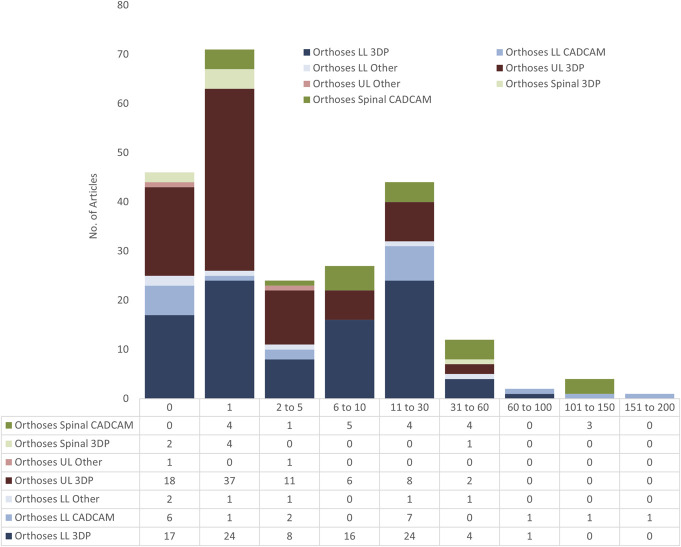
Number of LLO, ULO, and SO studies with a range of sample sizes.

Few studies had more than 5 participants, with most research having N = 0 or 1 (seen in Figure 12). Although this small scale is typical for immature technology, larger population samples are needed to move beyond individual bias and personal circumstances and provide generalizable results. The ethics of moving to larger scale trials is important to consider, and technologies should not be arbitrarily fast tracked to larger scales if this brings too much uncertainty and risk of injury.

The review identified a range of medium to larger sized cohort studies across LLOs, with 7 AFO articles having N > 10, 2 of which were N > 30, and 25 N > 10 articles concerning insoles, of which 7 were N > 30. Roberts et al (2016) conducted a N = 136 RCT comparing traditional casting with scan-based CAD/CAM.^29^ The findings were not positive, with no significant improvement in quality, cost, or time to delivery, and a higher incidence of fit problems.^29^

We note that with the advantage of lower risk for most insole studies, along with high numbers of potential commercial customers, insoles lead the way in terms of numbers of long-term trials with large cohorts, and justifying the large number of commercial digitally fabricated insole products on the market, using both CAD/CAM and AM. For AM insoles, Xu et al published studies with N = 80 ^22^ and N = 60,^23^ the former being a single blind randomized controlled study. For CAD/CAM insoles, which have been on the market for decades, 2 studies had 60 or more participants, both comparative studies with traditionally fabricated insoles. Govindasamya et al^26^ with N = 178 and Yurt et al^39^ with N = 67 conducted randomized control trials with significant favorable results in favor of the digitally fabricated products.

For AFOs, an interesting N = 50 study digitally mapped AFO manual fabrication.^7^ This modeling could be very helpful for informing development of new technologies or optimizing existing practice.

For the 13 ULO studies that have N ≥ 10, 6 had no outcome measures beyond a week. The 3 with N ≥ 20 are outlined below.

The largest trial had N = 60 and studied SLS-printed Nylon casts for forearm fractures, whose design was informed using FEA with computed tomography data.^31^ These were compared to plaster cast and splint fixation as control groups, with 20 patients in each group. Outcomes on clinical efficacy, wrist function, and patient satisfaction were scored and compared up to 3 months, with the printed group scores totaling higher than the other 2 groups. This trial was predated by the same group with a smaller, shorter N = 10 study with 6-week follow-up and patient satisfaction questionnaires^32^; however, this initial study did not involve FEA modeling. This is a good example of considered expansion of research protocol.

An N = 40 RCT compared SLA-printed wrist orthoses with conventionally fabricated orthoses after 6 weeks.^33^ Quantitative outcome measured such as the Modified Ashworth Scale showed no significant difference between groups; however, Fugl-Meyer Assessment and swelling scores showed significant change with the printed group vs. the control.^33^ Kim et al^40^ conducted a preliminary N = 22 randomized controlled open-label study on personalized wrist orthoses made with thermoplastic polyurethane (TPU) by FDM, for a period of 1 week.

SO had a range of larger studies, with 10 having N ≥ 30, all concerning TLSOs except one that looked at cervical collars. Four of these studies had N ≥ 100. The largest was a prospective controlled cohort observational study of 225 patients^35^ concerning the new Lyon brace or ARTbrace, which is an immediate corrective brace based on the same principles of previous plaster cast braces, but using CAD/CAM and existing proprietary software, OrtenShape. The article includes an in-detail description of the design process and theory of action. The ARTbrace showed better Cobb angle reduction rates and improved aesthetic appearance.^35^

A comparative trial on 147 people with AIS included 43 people using the conventional method and 104 using the CAD/CAM method.^6^ Cobb angle, apical vertebral rotation, and trunk listing were measured at prebrace, 4, 8, and 12 months postbrace provision. They found significant decreases (*p* < 0.05) in the Cobb angles when compared to preintervention data. The mean productivity of the CAD/CAM method was 2.75 times higher than that of the conventional method, however, with learning curve potentially requiring 4 years to achieve this. The CAD/CAM method could achieve similar clinical outcomes with high efficiency and be used as a substitute for conventional methods in fabricating these spinal orthoses.

A single-center prospective randomized controlled trial also looked at AIS with N = 120, and 94 people completing the whole study of 2 years.^38^ Orthoses designed using CAD/CAM compared applications with and without patient-specific FEM simulations of the spine, rib cage, and pelvis. Results were not significantly different, with satisfying outcomes in both groups.^38^

D'Amato et al (2001) conducted an N = 102 trial on specifically female patients with AIS, with excellent in-brace correction observed with the CAD/CAM braces.

### Qualitative methods

**Figure 13.**
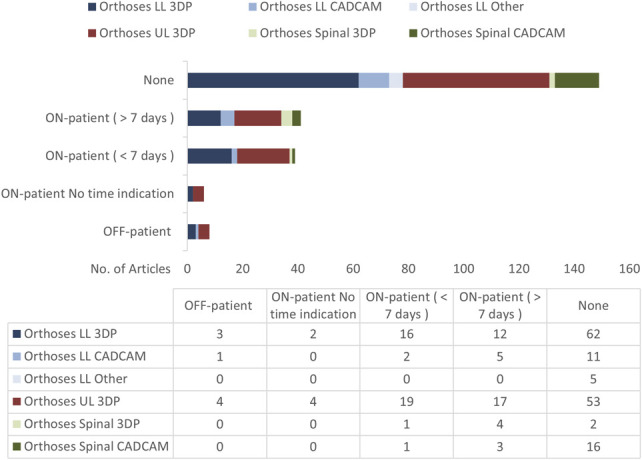
Number of LLO, ULO, and SO articles with qualitative methodologies. ON-patient refers to measures performed when the tested device is in situ and in use on a person. OFF-patient refers to when the tested device is not on a person.

The number of studies using qualitative methods are shown in Figure 13. Most articles do not include qualitative evaluation of the outcomes. This likely reflects the intention of the study and maturity of technology being researched. That said, user involvement/patient and public involvement and engagement is now a prerequisite for quality research.

For LLO CAD/CAM, 5 articles used qualitative methods beyond a week. The only AFO study in these was Roberts et al^29^ for their N = 150 over 12 months on AFOs used. Patient-focused outcome measures included the “Satisfaction with Device” and “Satisfaction with Service” questionnaires from and Orthotics and Prosthetics Users' Survey (OPUS), which use Likert-type scales. The questions were administered by post at 3-, 6-, and 12-month follow-up. After experiencing both casting and scanning (in random order), 70% of the patients said they preferred being scanned to having the limbs cast in plaster. A Mann–Whitney U test to evaluate differences found no significant differences between the allocated groups. The overall results did not support the introduction of CAD/CAM-based AFOs.

The 4 insole studies were by Govindasamya et al,^26^ Yurt et al,^39^ Shojaie et al,^41^ and Zwaferink et al.^42^ Out of these, Yurt et al^39^ is most notable here with an N = 67 study on people with painful flexible flatfoot (PFFF). They looked at pain intensity and quality of life measures using the Short Form-36 and the International Physical Activity Questionnaire-Short Form to assess activity levels. Participants were also asked to rate their insole satisfaction using a visual analog scale. Ultimately however, they found no between-group differences in terms of the initial assessment of pain intensity, foot function index, and health-related quality of life for the CAD/CAM vs conventional insoles provided.

LLO 3DP had 12 articles with qualitative evaluation over a week. Ten of these were insole studies, with 4 providing N>20.^22-25^ A notable example is Tarrade et al who asked 34 standing workers who experience foot pain to assess pain and comfort levels in using a 3D-printed insole, using the Foot Health Status Questionnaire. The questionnaire assesses foot health during the past week in terms of pain (type, intensity), foot function (walking, working), footwear, and general foot health. The results found a significant decrease in pain and perception of general foot health from use of the prototype insoles.^24^

For 3DP AFOs, only 2 studies evaluated qualitative outcomes beyond a week. Meng et al (2021)^43^ used Numerical Rating Scale (NRS) scoring on 15 participants across material comfort, weight feeling, surface smoothness, difficulty in wearing, convenience of cleaning, skin lesion, and the occurrence of adverse events; this is discussed in the Materials Section. Deckers et al^44^ made an AFO design using SLS and tested on 7 participants with data collected up to 7 weeks. An interesting and clear break down of the mechanical failures was provided for all 7. The control set of traditionally fabricated control AFOs did not report any issues over the test period, indicating the clear need for design optimization.

For ULO 3DP, 17 articles qualitatively evaluated their technologies beyond a week across a range of TRL levels and samples sizes. Only 3 were above N = 20. Chen et al,^31^ N = 60, compared SLS forearm fracture casts with outcomes on clinical efficacy, wrist function, and patient satisfaction for up to 3 months, with favorable results. Zheng et al^33^ reported a RCT on SLA-printed WOs with a 6-week duration and with a range of measures including the Quebec User Evaluation of Satisfaction with Assistive Technology (QUEST) assessment to evaluate the participant satisfaction when wearing the orthoses. The printed orthoses scored better than the control thermoplastic orthoses for functional outcomes, but no significant difference in these qualitative measures. Kim et al^40^ conducted a preliminary N = 22 randomized controlled open-label study on wrist orthoses made with FDM, with The Patient-Rated Wrist Evaluation and OPUS were checked before and 1 week after the application, with varying differences between the FDM and control groups.

For SO CAD/CAM, only 3 articles collected long-term qualitative data.^34,38,45^ Notable here is Guy et al (2021) with an N = 120 study on patients with AIS, using the SRS-22r Quality of Life outcome questionnaire over 2 years among other physical outcome measures, finding no significant difference in these outcomes for the CAD/CAM group over the control group.^38^

For the 7 SO 3DP studies, 4 had qualitative outcomes beyond a week^46-48^; however, only Kuo et al^30^ had more than 1 participant, with N = 41, with simple comfort scoring - they found higher scores for their customized cervical collars.

Although the high numbers of articles focusing only on OFF-patient evaluation is appropriate, the paucity of ON-patient data beyond a week of use severely limits the community's ability to understand these technologies' true value and potential impact, creating a barrier to downstream implementation. In reality, less than a week generally refers to no evaluation outside the lab or clinic. Studies over 4 months are needed to adequately assess outcomes, but these are entirely missing from the literature for nearly all device categories. Without this formal evidence, it is extremely difficult to make informed, justified decisions on the use of these technologies, or the future direction that they should take.

### Quantitative methods

**Figure 14.**
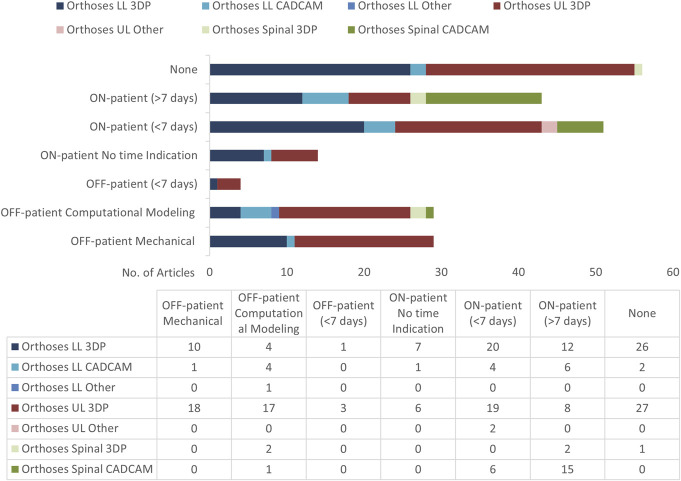
Number of LLO, ULO, and SO articles with quantitative methodologies. ON-patient refers to measures performed when the tested device is in situ and in use on a person. OFF-patient refers to when the tested device is not on a person.

Quantitative outcome measure data are important for its more objective nature and to enable easy communication of technology efficacy to both the orthotics community and wider audiences such as funders and policy makers. The number of studies using quantitative methods are shown in Figure 14, for example the Cobb angle, which is the most widely used measurement to quantify the magnitude of spinal deformities. Quantitative analysis of qualitative participant evaluation scales, such as comfort, is only meaningful when there are large enough numbers to counter both individual physical response differences in the body, or individual differences in very subjective perceived characteristics of device outcomes.

For LLO CAD/CAM, 6 articles used quantitative methods beyond a week, 5 concerned insoles.^26,29,39,41,42^ A range of quantitative measures for insole evaluation are applied, for example Govindasamy et al (2020)^26^ looked at ulcer recurrence for 178 patients, and multiple studies use pressure mapping technologies to evaluate outcomes, for example Zwaferlink et al^42^(2020) looking at metatarsal head peak pressure compared with nontherapeutic shoes, finding no significant difference between footwear concepts.

In LLO 3DP, there are 10 articles looking at insoles.^22-25,49-54^ These use a range of quantitative methods specific to insoles, in particular plantar pressure mapping of the foot, for which there is many commercial systems available, and various using gait analysis. The largest 2 studies are from Xu et al with mature studies of N = 80 and N = 60.^22,23^ For example, the N = 60 study on patients with plantar fasciitis used the Footscan® system recording maximum pressure, maximum strength, and contact area of patients’ hallux, toes 2-5, first to fifth metatarsal, midfoot, lateral heel, and midfoot heel at weeks 0 and 8, as well as visual analogue scale scores at weeks 0 and 8 to assess overall comfort of foot orthosis, to determine the credibility and comfort of the orthopedic insole conditions. The results supported the efficiency of customized 3D printing AFOs over prefabricated versions for reducing damage associated with plantar lesions.

For 3DP AFOs, there are the only 2 with any quantitative measures beyond a week. Meng et al (2021)^43^ discussed below in the materials section, and Deckers et al (2017)^44^ with 7 participants testing laser-sintered AFOs, only looking at fit time and observational individual device outcomes on breakage over 6 weeks, with 6 out of 7 devices not lasting this period.

For ULO 3DP, there were 19 articles that have longer term quantitative data, but with just 3 above N = 20—Zheng et al (2020)^33^’s RCT with a notably strong range of established outcome measure protocols including the Modified Ashworth Scale and the Fugl-Meyer Assessment over 6 weeks, Chen et al (2020)^31^ with N = 60 with assessments of clinical effectiveness and patient satisfaction over 3 months, and the Cooney modification of the Green and O'Brien score applied for the wrist functional assessment after 3 months, including the evaluation of pain, functional status, range of motion, and grip strength. Kim et al^40^ (2018) applied the Jebson Hand Function Test at 1 week.

For SO CAD/CAM, there is a large range of quantitative datasets available. Fifteen articles (out of 22 articles overall) collected long-term quantitative data, 9 have N > 20, 4 have N > 100, Mauroy et al (2014),^35^ Wong et al (2006),^6^ and Guy et al (2021).^38^ For example, Wong et al (2006)^6^ with N = 147 measuring Cobb angle, apical vertebral rotation, and trunk listing, giving highly useful evidence in favor of the CAD/CAM devices, and other large study size articles providing a similarly strong evidence base for this device category in favor of the use of CAD/CAM-based spinal braces.

For SO 3DP, there is very limited long-term data, only Kuo et al (2019)'s cervical collar study^30^ gathered datasets on more than 1 participant, with N = 41 measuring head neck and trunk angles in various body positions, finding improved effects with their customized collars.

An entire category that could be of great utility does not appear at all in the literature, ON-patient long-term mechanical testing. This could entail the re-evaluation of a device’s structural integrity and various material properties after a prolonged period of use and shed light on the key question of device durability.

### Materials used

**Figure 15.**
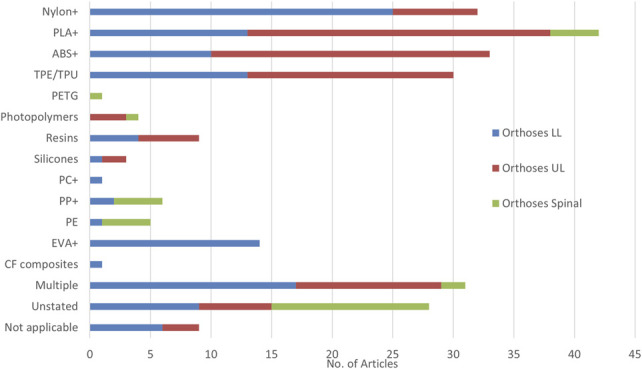
Materials used for the final product investigated in articles for LLO, ULO, and SO.

Across articles, more than 60 distinct materials were used for the primary construction. In Figure 15, these are grouped to analyze the data with a “+” denoting the inclusion of various composites of the given material. Photopolymers and resins contained a wide range of proprietary materials. A few noteworthy trends arise from this. First, a large number of articles do not state the material used, which is problematic for most categories; however, this is less of a problem for SO CAD/CAM articles that contribute strongly to this number because the mold is digitally produced and traditional materials and methods form the orthosis.

Second, the literature is dominated by Nylon, ABS, and PLA derivatives, rather than the materials from conventional workflows such as polypropylene and fiberglass. The main reason for this is driven by their suitability for AM processes rather than their material properties in application. The manufacturing process dictates much of the resultant material properties. For example, FDM-extruded polypropylene is not equivalent to vacuum-formed sheet polypropylene. The material properties of AM-produced devices are one of the most talked about concerns around the technology, with strength and durability being most in question. Meng et al^43^ used 3 different materials: PA2200, printed with SLS and characterized by high strength, light weight, and toughness; Somos NeXt, printed with SLS and with high strength and toughness and good precision and appearance; and PA12, printed with MJF and having extremely low moisture absorption, excellent mechanical strength, and good wear resistance and corrosion resistance.^43^ Differences were found in patient experience across a range outcomes, with Somos NeXt being the most popular.^43^ Gόrski et al^55^ investigated in detail the properties of common FDM materials (ABS, PLA, PA12, High Impact Polysterene [HIPS]) in regard to print quality across a number of parameters including cost, print quality, and infills. The study analyzed these across a range of combinations, with the study focused on wrist orthoses. The ABS and PLA performed generally well; the HIPS, however, did not perform well, and notable is that high percentage infills gave inconsistent results across testing. They recommended that PLA is a material that could be considered for medical products via 3D printing and maintaining environmental friendliness; however, only for low dynamic load or low temperature, chemical-free environments—if this is not the case, then ABS is recommended.

### Chronology

**Figure 16.**
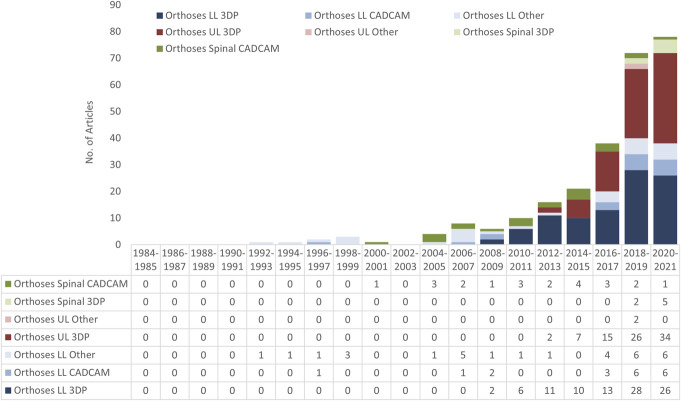
Number of LLO, ULO, and SO articles by year.

Figure 16 looks at the chronology of articles published on digital fabrication of orthoses. We see a rapid rise overall in recent years. It should be noted that the 2020–2021 totals would be larger; however, data collection was done in the first half of 2021. There were particularly rapid recent rises in the research being done in ULP and LLP 3DP. Although numbers were small, it was interesting that the spinal CAD/CAM articles were fairly consistent from 2004 to 2021, with 1–4 articles being published, and spinal 3DP's 7 articles all published since 2018.

## Discussion

Digital manufacturing is a transformative progression for orthotics practice. The evidence presented in this review shows that a large range of device types and manufacturing requirements can benefit from digital manufacturing. However, the research is at differing evidence levels, indicating gaps in the evidence that can be used for clinical and technical decision-making.

Extensive evidence was developed for insole technology across fabrication processes, incorporating CAD/CAM and AM. An advantage with insoles is that long-term testing can present lower risk to patients for some applications, whether highly experimental or mature in development. This is not to say that clear risks are not present for insoles universally, however, with neuropathy related risks and ulcer exacerbation, particularly with diabetic patients giving the possibility of leading to amputation. Insoles created through various digital means have been commercially available for some time, including AM-produced products. Several reasons explain the good evidence for this product family. Foot orthoses can be milled with a small 3-axis machine or printed with a small 3D printer, or printed in bulk with larger machines, making the technology digital manufacturing equipment accessible and lower cost. Foot orthoses is likely the largest device category, with many potential research participants across multiple clinical facilities (orthotics, pedorthics, podiatry, etc). From a design perspective, a lower tensile strength needed is for successful products, and many foot-specific CAD products are available for clinicians. Blind studies are more easily achievable for foot orthoses, enabling user blinded randomized control trials, unlike other orthoses where the manufacturing approach will not be concealable.

Multiple long-term trials up to 3 years were found for TLSO studies using CAD/CAM, reaching far back in the chronology. The labor savings to be found using digital techniques for orthoses are high because of the surface areas involved for casting. For example, Wong et al (2006) showed that productivity increased by 275% with CAD/CAM techniques for SOs; however, clinicians took up to 4 years to fully achieve this level.^6^ Quantitative measures are more common for scoliosis bracing than other devices in the literature, possibly because diagnostic imaging are typically ordered by the physician to monitor scoliosis progression while diagnostic images are typically not available for other orthotic device treatments. TLSO CAD/CAM articles usually had full descriptions of the production processes, compared to other orthosis research. CAD/CAM’s strong use in current clinical practice for TLSOs has likely been enabled by the development of this strong evidence base.

Across other orthosis types, training approaches and process shifts needed when switching to digital methods are not well discussed. This would be of great benefit to the other orthosis categories. Much of this training would be in the realm of continuing professional development (CPD), which may partially explain why research has been lacking since few CPD studies exist in the prosthetics and orthotics field. In addition, process shifts may remain business-related knowledge that private facilities do not share with competitors. Research on how clinicians use digital design software, how digital production is employed (central fabrication, staff and time for in-house carving or printing, etc), and long-term follow-up for device repairs would help the field move forward more efficiently. This research could include fuller mapping of the traditional processes across device types. This has been done in some cases, with an excellent example by Wang et al (2021)^7^ who digitally analyzed the traditional manual processes for pediatric ankle-foot orthoses. This team represent the Printhotics project, which continue to contribute very useful work.^56^

For AM devices, most studies were on devices with less risk to participants, devices where limited weight or force will be exerted (avoid high tensile stress), and insoles that have full body weight applied but mainly with compression/bending in the applied print plane and a smaller build volume. Other categories that present strong evidence were AM-produced wrist orthoses and immobilizing casts, with some statistically significant datasets from larger trials provided by Zheng et al^33^ and Chen et al.^31^ It was noted that Chen et al's team preceded their N = 60 trials with a similar N = 10 a few years previously.^57^ We found few other examples in the literature of where groups scale up their studies in this methodical way. This may suggest that obtaining funding or motivation for mid-scale trials is limited.

Capturing the lower limb and modifying the resulting digital model remains an obstacle for widespread implementation. Since the foot needs to be positioned during digitization, scanning becomes problematic. Software had advanced in this area, but research on obtaining a viable lower limb model and then correcting the pathological limb orientation in software before designing the lower limb orthosis is lacking and deserves attention to guide clinical practice.

The last decade's large acceleration of literature on digital fabrication methods in orthotics shows no sign of abating, but the point when concrete evidence will be available for many device categories is unclear. What cannot be captured in this review is the more general, product-agnostic development and expansion of the range of AM technologies and what capabilities are on offer. With many other major influences driving innovation, such as the automotive, military, and aerospace industries, newer AM technologies will likely overcome current limiting factors, such as tensile and torsional strength, print times, and infrastructure costs. Although the specifications of these new technologies are unlocking barriers, it is human behaviors and choices to change current practices, adopt new opportunities, respond to new knowledge and training, and use devices delivered through new processes that ultimately determines the success or otherwise of digital fabrication.

The rapid pace of AM advancement also is an inhibiting factor for traditional high quality research, such as RCT. The time required to create, fund, recruit, complete, and publish a RCT could result in a publication that is no longer relevant because of advancements in technology or processes. Other research methods that integrate outcome measurement into clinical practice, small case studies, and long-term multi-center collaboration for research data collection should be considered to expand the evidence base. Publication of failed approaches would also benefit clinicians and managers, since many orthotics facilities are likely repeating poor practice as they begin to apply digital manufacturing or change their digital approaches as part of continuing quality improvement.

## Summary

- Strong evidence exists for CAD/CAM technologies applied to spinal orthotics and insoles, and these study designs should be used as a guiding light for other application areas across digital fabrication.
- The evidence base is dominated by quantitative measures related to devices and processes, and far less the experiences of those involved in the production workflows, those dispensing orthoses and end users. All stakeholders are required to be beneficiaries of technology if any process or device is to deliver the potential advantages it proposes.
- Qualitative data are generally lacking across all orthoses areas and would allow much deeper understanding of the benefits or pitfalls of digital technologies and the ultimate effect they have for users.
- There is limited research literature available on the training of personnel and effect on performance. However, for some established areas, like CAD/CAM of TLSOs, there is good evidence of strong productivity benefits, albeit with long skills development times. Research investigating is needed across more areas to support clinics in decisions on uptake.
- Strong evidence for AM is more limited but is available for some device types—insoles and wrist orthoses in particular. Data suggest that AM could be appropriate across many orthosis areas, although further validation is still needed.
- The amount of research being published using digital fabrication in orthotics is rapidly accelerating; however, the bulk remain at more immature levels of technology development – mature, long-term, larger scales trials are still lacking for many areas.

## Limitations

Although the search strategy was comprehensive and the community at ISPO World Congress 2021 contributed by checking the body of literature for missing articles, there will inevitably be articles that have been missed from this review. For some topics of enquiry, there is a degree of subjectivity in the designations.

## Conclusion

Although some areas of digitally fabricated orthotics have strong evidence pertaining to their efficacy, such as insoles and spinal braces, which has enabled their ubiquitousness in clinical practice, evidence for most areas is lacking. For systems such as these that are working well, clearer ways to integrate digitization into clinics are needed. As well as product integrity, further research into the training requirements, appropriate dissemination of knowledge, and resultant real world productivity gains is needed to support decision makers in individual clinics or health services worldwide. It is also noted that evidence that better incorporates real world follow-up and assessment of failures would be highly beneficial. Further collaboration between academia, industry, and clinical teams across more of the pathway to market for new technologies could be a route to addressing these gaps, and the next steps on achieving this should be brought to the orthotics community for further discussion.

## Funding

This research was funded by Foreign, Commonwealth and Development Office (FCDO, formerly Department for International Development (DFID)), grant number GB-GOV-1-300815 Award date: January 28, 2019.

## Supplemental material

Supplemental material for this article is available in this article. Direct URL citation appears in the text and is provided in the HTML and PDF versions of this article on the journal’s Web site (www.POIjournal.org).

## Declaration of conflicting interest

The author(s) declared no potential conflict of interest with respect to the research, authorship, or publication of this article.

## Supplementary Material
