# Supplementary material for: A scoping review of digital fabrication techniques applied to prosthetics & orthotics: Part 2 of 2—orthotics
Source: Prosthet Orthot Int. 2024 Nov 13;49(4):427–44. doi: 10.1097/PXR.0000000000000399 (PMC12329800; doi:10.1097/PXR.0000000000000399)
Supplement: Supplementary file 1 [file poi-49-427-s001.docx]

**Appendices**

**Appendix 1: Acronyms used in this article**

AFO – Ankle Foot Orthosis

TLSO – Thoracic Lumbar Spinal Orthosis

LLO – Lower limb Orthosis

ULO – Upper limb Orthosis

SO – Spinal Orthosis

AIS – Adolescent Idiopathic Scoliosis

AM/3DP – Additive Manufacture / 3D Printing

CAD/CAM – Computer Aided Design/ Computer Aided Manufacture

CNC – Computer Numerical Control

FDM – Fused Deposition Modelling

SLA – Stereolithography

MJF – Multi Jet Fusion

ABS – Acrylonitrile Butadiene Styrene

EVA – Ethylene Vinyl Acetate
PLA – Polylactic Acid

HIPS – High Impact Polystyrene

TPU – Thermoplastic Urethane

TRL – Technology Readiness Level

CPD – Continued Personal Development

FEA / FEM – Finite Element Analysis / Modelling

RCT – Randomised Control Trial

OPUS – Orthotics and Prosthetics Users’ Survey

FHSQ – Foot Health Status Questionnaire

NRS – Numerical Rating Scale

QUEST – Quebec User Evaluation of Satisfaction with Assistive Technology

**Appendix 2: Example Search String**

(prosthe* OR amput* OR “residual limb” OR “residual limbs” OR “artificial limb” OR “artificial limbs” OR “artificial arm” OR “artificial arms” OR “artificial elbow*” OR “artificial extremit*” OR “artificial knee” OR “artificial knees” OR “artificial leg” OR “artificial legs” OR “artificial shoulder” OR “artificial shoulders” OR “bionic arm” OR “bionic hand” OR disarticulation OR rotationplasty OR "rotation plasty" OR rotation-plasty OR "Van Nes" OR "Van Nes’s" OR "Syme’s" OR orthotic OR orthosis OR orthoses OR brace OR bracing OR "orthopaedic device" OR splint OR AFO OR KAFO OR HKAFO OR TLSO OR LSO OR exoskeleton OR "activity suit " OR insole OR in-sole OR insoles OR scoliosis OR "spinal cast")

AND

("3D print*" OR "three dimensional print*" OR "3 Dimensional print*" OR "3D fabricat*" OR "three dimensional fabricat*" OR "3 Dimensional fabricat*" OR FDM OR "fused deposition modelling"  OR SLA OR stereolithography or SLS OR "laser sintering" OR "laser melting" OR "multi jet fusion" OR "digital manufactur*" OR "digital fabricat*" OR "additive manufactur*" OR "computer-aided manufactur*" OR stereolithography or CNC OR CADCAM OR "CAD/CAM" OR "CAD CAM" OR CAD OR "digital transformation" OR "layered manufactur*" OR "electron beam melting" OR "3D prototyp*" OR "three dimensional prototyp*" OR "3 Dimensional prototyp*" OR "rapid manufactur*" OR "rapid prototyp*" OR "rapid fabricat*" OR "freeform fabricat*" OR polyjet OR milling OR rectification

NOT (joint OR dental OR facial OR maxillofacial OR maxillary OR maxilla OR craniofacial OR denture OR dentures OR overdenture OR overdentures OR implant OR implants OR oral OR nasal OR cardiovascular OR vertebral OR vertebra OR "radial head" OR periprosthetic OR periprosthetic OR peri-prosthetic OR peri-prosthetics OR mandibular OR mandible OR condylar OR ocular OR scaphoid OR scapular OR scapula OR pelvic OR talar OR speech OR obturator OR tantalum OR orbital OR penile OR pubic OR maxillectomy OR pedodontic OR cartilage OR Miller-Galante OR periodontal OR sternal OR bladder OR orthodontic OR dentistry OR auricular OR tumor OR tumors OR tumour OR tumours OR ear OR tooth OR cleft OR sacrum OR scleral OR crown OR crowns OR talus OR cranial OR zirconia OR resection OR cranioplastic OR cranioplasty OR sternal OR arthroplasty OR calcaneal OR periodontally OR aortic OR aorta OR retinal OR osteotomy OR heart OR rhinoplasty OR nose OR orofacial OR rib OR edentulous OR bone OR jaw OR canal OR dentition OR cardiac OR endocarditis OR thrombosis)

**Appendix 3: Overview of articles specifically discussed in this review (nb. not all articles selected for review are discussed in detail in the narrative of this review)**

| Device Category | Article Reference | Year | **Device Body Location** | **Digital Manufacturing Method** | **Fabrication Workflow** | **Stated Materials for final manufactured component** | **TRL Level** | **N=** | **Quantitative Testing Methodology** | **Qualitative Testing Methodology** |
| --- | --- | --- | --- | --- | --- | --- | --- | --- | --- | --- |
| Orthoses Lower Limb | Meng et al.[40] | 2021 | Ankle-Foot | Multiple | PhysioCADAMMP | Multiple | TRL3 | 15 | ON-patient (<7 Days) | ON-patient (>7 Days) |
| Orthoses Lower Limb | Wang et al.[6] | 2021 | Ankle-Foot | Modeling (FEA or similar) only | No manufacturing took place | Not applicable | TRL6 | 50 | OFF-patient Computational Modeling | None |
| Orthoses Lower Limb | Govindasamya et al.[23] | 2020 | Insole | CAD/CAM (CNC based) | PhysioCADCNCPA | EVA (Ethylene-Vinyl Acetate) | TRL8 | 178 | ON-patient (>7 Days) | ON-patient (>7 Days) |
| Orthoses Lower Limb | Khodaei et al.[24] | 2017 | Insole | CAD/CAM (CNC based) | PhysioCADCNCPA | Unstated | TRL9 | 19 | On-patient No time Indication | None |
| Orthoses Lower Limb | Ki et al.[25] | 2008 | Insole | CAD/CAM (CNC based) | PhysioCADCNC | Unstated | TRL9 | 30 | On-patient (<7 Days) | None |
| Orthoses Lower Limb | Roberts et al.[26] | 2016 | Ankle-Foot | CAD/CAM (CNC based) | PhysioCADCNCPA | Polypropylene | TRL8 | 136 | ON-patient (>7 Days) | OFF-patient (>7 Days) |
| Orthoses Lower Limb | Shojaie et al.[38] | 2020 | Insole | CAD/CAM (CNC based) | PhysioAlgAMP | EVA (Ethylene-Vinyl Acetate) | TRL5 | 14 | ON-patient (>7 Days) | ON-patient (>7 Days) |
| Orthoses Lower Limb | Yurt et al.[36] | 2019 | Insole | CAD/CAM (CNC based) | PhysioCADAMP | EVA (Ethylene-Vinyl Acetate) | TRL6 | 67 | None | On-patient (>7 Days) |
| Orthoses Lower Limb | Zwaferink et al.[39] | 2020 | Insole | CAD/CAM (CNC based) | PhysioCADClinCNCP | EVA (Ethylene-Vinyl Acetate) | TRL6 | 24 | On-patient (>7 Days) | On-patient (>7 Days) |
| Orthoses Lower Limb | Salles et al.[22] | 2013 | Insole | AM-SLS/MJF | PhysioCADAM | Nylon-12 | TRL6 | 38 | ON-patient (>7 Days) | ON-patient (>7 Days) |
| Orthoses Lower Limb | Tarrade et al.[21] | 2019 | Insole | AM-SLS/MJF | PhysioCADClinAMPA | Polyamide 12 (PA 12) | TRL6 | 34 | ON-patient (>7 Days) | ON-patient (>7 Days) |
| Orthoses Lower Limb | Belokar et al.[41] | 2017 | Ankle-Foot | AM-FDM/FFF | PhysioCADAM | ABS (acrylonitrile butadiene styrene) | TRL3 | 1 | OFF-patient Mechanical | None |
| Orthoses Lower Limb | Xu et al.[20] | 2019 | Insole | AM-FDM/FFF | PhysioCADAMP | EVA (Ethylene-Vinyl Acetate) | TRL8 | 80 | ON-patient (>7 Days) | ON-patient (>7 Days) |
| Orthoses Lower Limb | Xu et al.[19] | 2019 | Insole | AM-FDM/FFF | PhysioCADAMP | EVA (Ethylene-Vinyl Acetate) | TRL7 | 60 | On-patient (>7 Days) | On-patient (>7 Days) |
| Orthoses Spinal | D'Amato et al.[33] | 2001 | Thoracic-Lumbar-Sacral | CAD/CAM (CNC based) | PhysioCADCNC | Unspecified | TRL9 | 102 | ON-patient (>7 Days) | None |
| Orthoses Spinal | Guy et al.[35] | 2021 | Thoracic-Lumbar-Sacral | CAD/CAM (CNC based) | PhysioCADFEACNCPA | Unstated Conventional | TRL8 | 120 | ON-patient (>7 Days) | ON-patient (>7 Days) |
| Orthoses Spinal | Mauroy et al.[32] | 2014 | Thoracic-Lumbar-Sacral | CAD/CAM (CNC based) | PhysioCADCNCPA | Polypropylene | TRL9 | 225 | ON-patient (>7 Days) | None |
| Orthoses Spinal | Sankar et al.[31] | 2007 | Thoracic-Lumbar-Sacral | CAD/CAM (CNC based) | PhysioCADCNCPA | Polypropylene | TRL9 | 10 | ON-patient (>7 Days) | ON-patient (>7 Days) |
| Orthoses Spinal | Weiss et al.[43] | 2010 | Thoracic-Lumbar-Sacral | CAD/CAM (CNC based) | PhysioCADClinCNCMPA | Unstated | TRL5 | 1 | ON-patient (>7 Days) | ON-patient (>7 Days) |
| Orthoses Spinal | Weiss et al.[55] | 2015 | Thoracic-Lumbar-Sacral | CAD/CAM (CNC based) | PhysioCADFEACNCPA | Unstated | TRL5 | 21 | ON-patient (<7 Days) | None |
| Orthoses Spinal | Wong et al.[9] | 2006 | Thoracic-Lumbar-Sacral | CAD/CAM (CNC based) | PhysioCADCNCPA | Unstated | TRL8 | 147 | ON-patient (>7 Days) | None |
| Orthoses Spinal | Hale et al.[44] | 2020 | Cervical | AM-FDM/FFF | PhysioCADAMMPA | Nylon-12, Acrylic Styrene Acrylonitrile (ASA) | TRL5 | 1 | ON-patient (>7 Days) | ON-patient (>7 Days) |
| Orthoses Spinal | Kuo et al.[27] | 2019 | Cervical | AM-FDM/FFF | PhysioCADAMPA | PLA (polylactic acid) | TRL6 | 41 | ON-patient (>7 Days) | ON-patient (>7 Days) |
| Orthoses Spinal | Redaelli et al.[45] | 2020 | Thoracic-Lumbar-Sacral | AM-FDM/FFF | CompCADAMPA | PETG, PE, PP | TRL5 | 1 | OFF-patient Mechanical, ON-patient (>7 Days) | ON-patient (>7 Days) |
| Orthoses Upper Limb | Chen et al.[29] | 2017 | Wrist-Hand | AM-SLS/MJF | PhysioCADAMP | Nylon | TRL6 | 10 | ON-patient (>7 Days) | ON-patient (>7 Days) |
| Orthoses Upper Limb | Chen et al.[28] | 2020 | Wrist-Hand | AM-SLS/MJF | PhysioFEAAMP | Nylon | TRL7 | 60 | ON-patient (>7 Days) | ON-patient (>7 Days) |
| Orthoses Upper Limb | Górski et al.[53] | 2020 | Wrist | AM-FDM/FFF | PhysioCADAMMPA | Multiple | TRL4 | 1 | OFF-patient Mechanical | OFF-patient (<7 Days) |
| Orthoses Upper Limb | Kim et al.[42] | 2018 | Wrist | AM-FDM/FFF | PhysioCADAMPA | TPU (thermoplastic polyurethane) | TRL6 | 22 | ON-patient (>7 Days) | ON-patient (>7 Days) |
| Orthoses Upper Limb | van der Stelt et al.[46] | 2020 | Multiple Devices | AM-FDM/FFF | PhysioCADAMMP | PLA (polylactic acid) | TRL6 | 4 | None | ON-patient (>7 Days) |

**Appendix 4: Digital Workflows Key**

| **#** | **Designation** | **Scanning/Data** | **Rectification/ Modification** | **Computational optimization** | **Computational learning** | **Fabrication** | **Manual fabrication** | **Postprocessing** | **Final Adjustments** |
| --- | --- | --- | --- | --- | --- | --- | --- | --- | --- |
| 1 | **UAM** |  | Unspecified digital design alteration |  |  | Automated AM of product |  | No substantial postprocessing | No final adjustments |
| 2 | **CADAM** |  | Digital design altered with manual CAD work |  |  | Automated AM of product |  | No substantial postprocessing | No final adjustments |
| 3 | **CADAMPA** |  | Digital design altered with manual CAD work |  |  | Automated AM of product |  | Post processing, assembly and addition (with alignment) of other components | Final adjustments / corrections before & during gait or equivalent training until comfortable |
|  | **CADCLAM** |  | Digital design altered with manual CAD work |  | Computational learning | Automated AM of product |  | No substantial postprocessing | No final adjustments |
| 4 | **CADISF** |  | Digital design altered with manual CAD work |  |  | Automated Incremental Sheet Forming of product |  | No substantial postprocessing | No final adjustments |
| 5 | **CADCNC** |  | Digital design altered with manual CAD work |  |  | Automated CNC/Milling of product |  | No substantial postprocessing | No final adjustments |
| 6 | **CADFEAAM** |  | Digital design altered with manual CAD work | FEA to optimise final design |  | Automated AM of product |  | No substantial postprocessing | No final adjustments |
| 7 | **CADFEAAMPA** |  | Digital design altered with manual CAD work | FEA to optimise final design |  | Automated AM of product |  | Post processing, assembly and addition (with alignment) of other components | Final adjustments / corrections before & during gait or equivalent training until comfortable |
| 8 | **CADFEACNCP** |  | Digital design altered with manual CAD work | FEA to optimise final design |  | Automated CNC/Milling of product |  | Post processing, assembly and addition (with alignment) of other components | No final adjustments |
| 9 | **CompCADAMPA** | Scan of component | Digital design altered with manual CAD work |  |  | Automated AM of product |  | Post processing, assembly and addition (with alignment) of other components | Final adjustments / corrections before & during gait or equivalent training until comfortable |
| 10 | **AnatFEAAM** | Complex Anatomical data collection |  | FEA to optimise final design |  | Automated AM of product |  | No substantial postprocessing | No final adjustments |
| 11 | **AnatFEAAMP** | Complex Anatomical data collection |  | FEA to optimise final design |  | Automated AM of product |  | Post processing, assembly and addition (with alignment) of other components | No final adjustments |
| 12 | **AnatFEACNCP** | Complex Anatomical data collection |  | FEA to optimise final design |  | Automated CNC/Milling of product |  | Post processing, assembly and addition (with alignment) of other components | No final adjustments |
| 13 | **AnatCADAM** | Complex Anatomical data collection | Digital design altered with manual CAD work |  |  | Automated AM of product |  | No substantial postprocessing | No final adjustments |
| 14 | **AnatCADAMP** | Complex Anatomical data collection | Digital design altered with manual CAD work |  |  | Automated AM of product |  | Post processing, assembly and addition (with alignment) of other components | No final adjustments |
| 15 | **AnatCADAMPA** | Complex Anatomical data collection | Digital design altered with manual CAD work |  |  | Automated AM of product |  | Post processing, assembly and addition (with alignment) of other components | Final adjustments / corrections before & during gait or equivalent training until comfortable |
| 16 | **AnatCADAMMP** | Complex Anatomical data collection | Digital design altered with manual CAD work |  |  | Automated AM of product | Manual fabrication | Post processing, assembly and addition (with alignment) of other components | No final adjustments |
| 17 | **AnatCADAMMPA** | Complex Anatomical data collection | Digital design altered with manual CAD work |  |  | Automated AM of product | Manual fabrication | Post processing, assembly and addition (with alignment) of other components | Final adjustments / corrections before & during gait or equivalent training until comfortable |
| 18 | **AnatCADCNC** | Complex Anatomical data collection | Digital design altered with manual CAD work |  |  | Automated CNC/Milling of product |  | No substantial postprocessing | No final adjustments |
| 19 | **AnatCADCNCPA** | Complex Anatomical data collection | Digital design altered with manual CAD work |  |  | Automated CNC/Milling of product |  | Post processing, assembly and addition (with alignment) of other components | Final adjustments / corrections before & during gait or equivalent training until comfortable |
| 20 | **AnatCADFEAAM** | Complex Anatomical data collection | Digital design altered with manual CAD work | FEA to optimise final design |  | Automated AM of product |  | No substantial postprocessing | No final adjustments |
| 21 | **AnatCADFEACNCPA** | Complex Anatomical data collection | Digital design altered with manual CAD work | FEA to optimise final design |  | Automated CNC/Milling of product |  | Post processing, assembly and addition (with alignment) of other components | Final adjustments / corrections before & during gait or equivalent training until comfortable |
| 22 | **AnatCADClinAMPA** | Complex Anatomical data collection | Digital design altered with manual CAD clinically informed |  |  | Automated AM of product |  | Post processing, assembly and addition (with alignment) of other components | Final adjustments / corrections before & during gait or equivalent training until comfortable |
| 23 | **AnatCADClinFEAAMPA** | Complex Anatomical data collection | Digital design altered with manual CAD clinically informed | FEA to optimise final design |  | Automated AM of product |  | Post processing, assembly and addition (with alignment) of other components | Final adjustments / corrections before & during gait or equivalent training until comfortable |
| 27 | **AnatCADClinCNCP** | Complex Anatomical data collection | Digital design altered with manual CAD clinically informed |  |  | Automated CNC/Milling of product |  | Post processing, assembly and addition (with alignment) of other components | No final adjustments |
| 29 | **AnatCADClinCNCMPA** | Complex Anatomical data collection | Digital design altered with manual CAD clinically informed |  |  | Automated CNC/Milling of product | Manual fabrication | Post processing, assembly and addition (with alignment) of other components | Final adjustments / corrections before & during gait or equivalent training until comfortable |
| 31 | **ManCADAM** | Manual physiological data collection | Digital design altered with manual CAD work |  |  | Automated AM of product |  | No substantial postprocessing | No final adjustments |
| 33 | **ManCADAMMP** | Manual physiological data collection | Digital design altered with manual CAD work |  |  | Automated AM of product | Manual fabrication | Post processing, assembly and addition (with alignment) of other components | No final adjustments |
| 36 | **ManCADCNC** | Manual physiological data collection | Digital design altered with manual CAD work |  |  | Automated CNC/Milling of product |  | No substantial postprocessing | No final adjustments |
| 38 | **ManCADFEAAM** | Manual physiological data collection | Digital design altered with manual CAD work | FEA to optimise final design |  | Automated AM of product |  | No substantial postprocessing | No final adjustments |
| 39 | **ManCADClinFEAAMPA** | Manual physiological data collection | Digital design altered with manual CAD clinically informed | FEA to optimise final design |  | Automated AM of product |  | Post processing, assembly and addition (with alignment) of other components | Final adjustments / corrections before & during gait or equivalent training until comfortable |
| 40 | **ManCADClinCNCMPA** | Manual physiological data collection | Digital design altered with manual CAD clinically informed |  |  | Automated CNC/Milling of product | Manual fabrication | Post processing, assembly and addition (with alignment) of other components | Final adjustments / corrections before & during gait or equivalent training until comfortable |
|  | **Unclear** |  |  |  |  |  |  |  |  |
|  | **No manufacturing of orthosis/prosthesis took place** |  |  |  |  |  |  |  |  |

| **Key** | **Acronym** | **What it entails** |
| --- | --- | --- |
| Manual anatomical data collection | Man | Simple Manual measurements (ie.1D using ruler, tape, calipers etc), anthropometric databases |
| Complex anatomical data collection | Anat | Scan of body, Scan of a cast, Plantar Pressure, Subsurface, MRI, CT, Ultrasound (could also include additional 1D manual measurements) |
| Scan of component | Comp | A scan of a device component that has already been fabricated (likely for replication) |
| Digital design altered with manual CAD work | CAD | The design is altered/rectified with limited or no explanation of the reasoning |
| Digital design altered with manual CAD clinically informed | CADClin | The article gives some details of the design being clinically informed |
| FEA to optimise final design | FEA | Finite Element Analysis is used to optimise the final design of the device |
| Computational learning | CL | Machine Learning, artificial intelligence or other data science method |
| Automated CNC/Milling of product | CNC | Computer Numerical Controlled' Carving of the product |
| Automated AM of product | AM | Using any AM process, e.g. FDM, SLS, SLA, MJF, SLM |
| Automated Incremental Sheet Forming of product | ISF | Incremental Sheet Forming |
| Manual fabrication | M | Draping, Vacuum forming, casting |
| No substantial postprocessing | No acronym used | No postprocessing is described |
| Post processing, assembly and addition (with alignment) of other components | P | Post processing, assembly and addition of other components is discussed |
| No final adjustments | No acronym used | No final adjustments were done or they were not described |
| Final adjustments / corrections before & during gait or equivalent training until comfortable | A | Final adjustments / corrections before & during gait or equivalent training until comfortable were described |
